# An Account of the Opinions Recently Advanced by M. Magendie, with Regard to the Functions of Certain Nerves

**Published:** 1824-07

**Authors:** 


### APPENDIX.

### *An* ACCOUNT *of the* OPINIONS *recently advanced by* M. MAGENDIE, *with regard to the* FUNCTIONS *of certain* NERVES.

THE recent visit of M. MAGENDIE to London, and the public performance of his experiments on the fifth pair of nerves, have given to them a greater degree of interest than it is probable they would otherwise have excited, ingenious, and in some respects satisfactory, as they unquestionably are. On this account, we have hastened to lay the papers containing his new views upon this subject before our readers, and shall submit a few observations which their perusal, and witnessing a repetition of the experiments in question, have suggested: and if, in doing this, it shall appear that we differ from the author with regard to his conclusions, at the same time we are happy to bear testimony to the neat and skilful manner in which the experiments were performed, as well as to the solicitude with which he strove to render them intelligible and satisfactory to the gentlemen present. In the first place, we shall let M. Magendie speak for himself, giving his papers without any other alteration than this, that we have numbered the sections for the sake of referring to them more easily; and that we have placed in italics the most important, and (as it appears to us) most erroneous, of his conclusions.

"*Is the Olfactory Nerve the Organ of Smell?—Experiments on this question, by* M. MAGENDIE.

To ask if the olfactory be the nerve of smell, is it not to raise a laugh at the expence of the querist? Who doubts it? will be the answer. Has it not been an acknowledged truth, ever since anatomy made us acquainted with the disposition of the nerve,—its distribution on the olfactory surfaces,—its complete development in those animals which have the sense most perfect, &c. &c? I confess that, if the question had been put to me but last month, I should not have hesitated to answer it in the affirmative, nor should I have ventured to suggest a doubt upon the subject; notwithstanding that, in physiology, medicine, &c. it may not be without use to doubt somewhat, things which are the most certain: but let who wills, lay doubts aside.* Be this as it may: being desirous this season of demonstrating the different properties of the nervous system, I began with endeavouring to prove, by direct experiment, that the olfactory nerves bestow the sense of smell; an attempt which, so far as I know, had never hitherto been made.

1. My first experiment was to lay bare the olfactory nerves in a dog, about a year old. I scarcely expected to find them sensible to the contact of foreign bodies, or even to pricking, the hemispheres of the brain being insensible to such means of irritation throughout the greater part of their mass; and accordingly pressure, pricking them deeply, and tearing them in various ways, produced no effect which indicated sensibility of these nerves. I was curious to see whether the direct contact of a very odoriferous body would afford the same result:—I therefore poured some drops of ammonia upon the nerve; at first the animal seemed not to perceive it, but soon showed proofs of lively sensation. I immediaiely discovered that the liquid had flowed over the sides of the nerve, had reached its lower surface, and consequently was lodged in the pit of the ethmoid bone. I then was of opinion that the ammonia had acted on the medullary part of the nerve, which we know is expanded upon the cribriform plate, and that at least the white inferior surface of the nerve possessed sensibility, if the cineritious substance of the upper surface did not.

2. After these observations, I entirely destroyed the olfactory nerves, persuaded that I should then abolish completely the sense of smell. What was my surprise, on examining the animal the following morning, to find that he was yet sensible to powerful odours which I presented to him (such as ammonia, acetic acid, and the essential oil of lavender, &c.) The sensibility of the internal cavity of the nose had lost nothing of its energy: when a probe was introduced, the effects were exactly similar to those in a dog which had not been touched. This extraordinary phenomenon recalled to my memory a fact which I had passed over without much attention last year, because it stood so much opposed to received opinions, that I attributed the occurrence, I know not why, to some fault in the performance of my experiment. I allude to a duck, which survived for eight days after I had removed the hemispheres of the brain, presenting many curious phenomena. Among other singular circumstances, it still retained the power of perceiving strong odours. I exhibited this animal, and subjected it to various proofs of this, in the course I was giving at the time.

To be better satisfied of the fact, I destroyed the olfactory nerves in many other animals, and the results were exactly similar; but I made the important remark, besides, that the sensibility, which I had before observed at the inferior surface of the olfactory nerve, extended only along the outer border of the cribriform plate; and from this I was led to conjecture, that the sensibility might belong not to the nerve of smell, but to the branch of the ophthalmic which passes from the orbit into the nose through a fissure in the cribriform plate.

3. From this suggestion I was led to suspect, further, that the branches of the fifth pair, which are distributed among the cavities of the nose, are the organs by which the sense of smell is maintained after the destruction

* "Bien qu'en Physiologie, en Medicine, &c. il ne soit pas inutile de douter quelque peu des choses les plus certaines: mais ne doubte pas qui veut."

of the first pair of nerves. These branches in man are pretty numerous, though not very large; they consist; 1st, of the ethmoidal branch of the nasal: 2dly, the naso-palatin branch described by Scarpa: 3dly, of numerous branches which arise from the internal surface of the spheno-palatine ganglion. These nerves coincide in so far that they are all distributed upon the pituitary membrane.

I was not aware of the exact manner in which the fifth pair gives its branches to the nose of the dog; and I requested M. Desmoulins, who is well skilled in these researches, to dissect this nerve in the dog along with me. We discovered that the ethmoid branch is much larger than in man, and that it supplies a considerable number of small branches to the uppermost part of the nasal cavity; we found besides that there is no spheno-palatine ganglion on the superior maxillary division, but that it sends numerous branches of considerable size, which are distributed on the inferior, lateral, and internal parts of the nose.

4. It was then anatomically possible that the whole sensibility of the pituitary membrane might depend upon the divisions of the fifth pair. But conjectures concerning organic functions, derived from anatomy, are worth nothing until they are proved by physiological experiments. I therefore thought of cutting the fifth pair of nerves, in such a manner that the animals might survive. This was more easily said than done. The nerves, as they pass out of the base of the skull, are embraced by the cavernous sinus and the internal carotid artery. Nevertheless I made the attempt in several rabbits; and had the good fortune to succeed in cutting them on both sides, in a number of animals, without producing any very serious injury. I made the same attempt on puppies, kittens, and guinea-pigs, and, when the nerves were once well divided, I was able to assure myself that every trace of the action of powerful odours disappeared. The same animals, which, before the experiment, sneezed or rubbed their noses, or turned away their heads, when they were made to inhale ammonia or acetic acid, &c., continued perfectly undisturbed, when the fifth pair was divided, or only showed symptoms of the odour having affected the larynx. *The result of this experiment, in counterproof of the preceding, appears to me to shew, that the sense of smell, as far as regards powerful odours, depends upon the branches of the fifth pair: and that the first pair of nerves has no share with the fifth in bestowing this function.*

5. Here an objection presents itself. The odours employed, it may be said, are very active: they produce a chemical action upon the pituitary membrane just as when they come in contact with the conjunctiva. Is it not possible, when you destroy the sense of touch in the membrane of the nose, that you deprive it, not of the power of perceiving odours, properly so called, but rather of its sensibility to the impression of pungent and caustic vapours; such as those of ammonia and acetic acid? This remark may apply to these two vapours, but not to the oil of lavender or of dippel. At any rate, before the performance of these experiments, it could scarcely have been presumed, that irritating vapours did not act on the sense of smell.

6. For the purpose of doing away with this difficulty experimentally, I destroyed, by bruising them, (en les broyant) the olfactory nerves in a setter, the fineness of whose nose is well known; and I observed just as in my former experiments, that he could easily perceive powerful odours. But I was desirous of ascertaining whether he could distinguish the smell of meat, or cheese, or food in general. With this intention I enclosed portions of these in paper, and placed them before him; he invariably tore off the paper, and then devoured the contents. Yet I cannot regard this experiment as satisfactory, because the dog, in other circumstances, did not appear able to detect, by its smell, food placed near him without his knowledge. Supposing this last result to be correct, it does not prove, however, that the fifth pair is not the agent of the sense of smell, because the injury which is necessary to destroy the olfactory nerves, necessarily produces inflammation within the cavity of the nose, quite sufficient to injure, in a secondary way, the power of smelling. This subject I am pursuing at present. In chickens, ducks, and magpies, I removed the lobes of the brain with the whole of the olfactory nerves. They continued to possess all the sensibility which belongs to the membrane of the nose, and manifested evident signs of the action of powerful odours on the sense of smell: I cannot comprehend how the contrary has been lately published.

7. To M. Ramon, medical inspector of the Maison Royale at Charenton, I am indebted for a fact which appears to me to prove, that the integrity of the hemispheres of the brain is not indispensable for the exercise of smelling. It is common to see patients, who have been afflicted for several years with madness, fall suddenly into a condition of dullness and torpidity analogous to complete drunkenness; their limbs totter, their motions are not under their controul, and their speech is disturbed; this state, which is beyond the power of relief, is followed by an entire loss of the intellectual faculties, and not long afterwards by death. On examination, the hemispheres are found gorged with blood, the membranes inflamed, and the cortical substance deeply altered. M. Ramon has observed in such individuals that the sense of smell continued, not only with regard to powerful and pungent odours, but also to such as are much more transitory.

Such are the observations concerning the nerve of smell which I present to physiologists: they are as yet incomplete, and require to be followed up. I hope, however, that they may induce others to repeat them, and not to neglect an opportunity of confirming or invalidating them, by pathological observations.

8. From these researches it likewise follows, that animals, such as the dolphin, in which there are no olfactory nerves, are probably not deprived of the sense of smell, notwithstanding what has been asserted by naturalists. If it be confirmed that the faculty of smelling belongs to the fifth pair, we have still to enquire what can be the use of the olfactory nerves and their lobes. Nothing hitherto is known which is likely to point out the way to us, in which case they would require to be added to the list of those parts of the nervous system concerning the functions of which we are still entirely ignorant."

"*On the Influence of the Fifth Pair of Nerves on the Nutrition and Functions of the Eye.* By M. Magendie.

It has been seen, in the preceding Memoir, how I was led to cut the fifth pair of nerves within the cranium, in such a manner as not to destroy the life of the animal. I have thus been led to observe phenomena completely at variance with our received ideas respecting the functions of the nervous system.

After having cut the fifth pair of one side in a rabbit, I perceived that all sensibility was lost on the same side of the face; the inside of the nose, the surface of the conjunctiva, &c. being insensible to the contact of hard bodies, and even of sharp instruments. I wished to ascertain if the loss of sensibility also obtained with regard to very irritating chemical agents. For this purpose I applied ammonia to the eye; and I had no difficulty in discovering that it produced no impression. In order to have a test with which to compare it, I touched slightly the eye of the sound side with a little ammonia; and the animal immediately manifested (by its movements, its efforts, the abundance of tears, and closing the eyelids, &c.) the exquisite sensibility which is known to belong to the eye. There was nothing similar in the side where the nerve was divided: the eye was dry, and, what is extraordinary, the movement of the eyelids called winking had ceased; the ball of the eye itself appeared to have lost all motion; the iris was strongly contracted and immobile; in fine, the eye looked like an artificial one, placed behind eyelids which had no power of moving.

Much perplexed (*intrigué*) by the multitude of strange phenomena which I had observed, I delayed till next day the continuation of my researches, and I endeavoured to discover the rationale of what I had seen. The loss of sensibility of the surface of the eye, was the circumstance most easily understood; the distribution of the branches of the ophthalmic nerve to the eyelids, conjunctiva, &c. well explained this phenomenon; which, moreover, had been observed by Mr. Mayo on pigeons. The suspension of the secretion of tears might likewise be accounted for, by the paralysis of the lachrymal nerve; but the immobility of the eyelids, that of the eye, and the permanent contraction of the pupil, were not so easily reconciled with known facts. I contented myself with conjecturing that, in cutting the nervi trigemini, I had probably included the motores occulorum.

Next day I examined the animal, and was not a little surprised to find matters as I had left them: only the sound eye, in consequence of the application of the ammonia, was very violently inflamed; the other eye, on the contrary, presented no appearance of this kind. The section of the nerves had thus prevented the development of inflammatory action; and this result was not less curious than the preceding.

To enable me to study these various phenomena with care, I that day cut the fifth pair in a number of rabbits: in some, on one side; and in others, on both sides. It was by observing these animals during the following days, that I was led to discover the facts I am about to relate, and which will doubtless excite the interest of physiologists.

A. Twenty-four hours after the division, the cornea begins to become opaque, at the end of seventy-two hours it is much more so,—the opacity encreases, and five or six days after the operation it is as white as alabaster.

B. From the second day the conjunctiva becomes red, appears inflamed, and secretes a very abundant, milky, puriform matter: the eyelids are either wide open and motionless, or else they are sealed by the puriform matters which have dried between their edges, and when they are separated a considerable quantity of matter above described escapes.

C. Towards the second day after the section, the iris is likewise observed to become red, its vessels are developed, and, in fine, the organ inflames.—False membranes are formed on its anterior surface, which, like the iris itself, have the appearance of a disk pierced in the middle. These adventitious membranes at length fill the anterior chamber and contribute to the opacity of the cornea. Is it not a very extraordinary phenomena to witness an active inflammation with suppuration, and complete insensibility of the part inflamed, and which is caused by the division of a nerve? Before going farther, I would mention that this rapid opacity of the cornea at first appeared to me to depend upon the prolonged contact of the air. To ascertain this, I cut the seventh pair in a rabbit, which, according to Mr. Charles Bell, governs the movement of winking: but, although the eye of this animal remained constantly exposed to the air for many days, no opacity of the cornea took place; nor any inflammation either of the conjunctiva or of the iris. I then suspected that the opacity depended on the want of the secretion of tears. It is possible, said I, that a membrane such as the cornea may have occasion to be constantly soaked by a limpid fluid to maintain its transparency. To ascertain if my conjecture had any foundation, I completely removed the lachrymal gland in two rabbits, but no opacity of the cornea was perceptible during eight days that they survived this extraction. My supposition therefore was without foundation. The opacity of the cornea, the inflammation, and the suppuration of the conjunctiva and of the iris, were thus found to depend upon nervous influence.

D. Towards the eighth day after the section of the fifth pair the iris becomes visibly altered; it detaches itself from the sclerotica, and its centre ulcerates: at the end of two or three days the humours of the eye, being muddy and partly opaque, make their escape, and the eye is reduced to a small tubercle, which only occupies a very small part of the orbits, giving something hideous to the aspect of these animals. If the eye be now dissected, it is found to contain nothing but a matter which resembles cheese newly coagulated, and that the retina has almost entirely disappeared; a trace of it here and there is all that can be seen.

E. Vision appears to be, if not entirely lost by the division of the nerve, at least very much weakened, and if, some hours after the operation, a sharp instrument be pushed against the surface of the retina, the animal gives no mark of sensibility;—when both nerves are cut in an animal, he appears blind, and his gait is very singular: he only moves with the chin strongly pressed against the ground, pushing his head before him, and using it as a guide in the same way as a blind man does his staff. The conduct of an animal in this state differs entirely from that of one simply deprived of sight, the latter easily directing itself by means of the moustaches and of the sensibility of the skin of the face: he stops before holes, perceives obstacles,—in short, it would often be difficult to know whether he was blind or not. Whilst the animal in whom the fifth pair has been divided has only one way of moving, and instead of avoiding obstacles, often persists in pushing against them for many hours so as to excoriate the skin on the fore part of the head.

F. The tongue is insensible on the side where the nerve is cut, and on either side if both nerves be divided. The animal in this case holds it out of its mouth, but is able to draw it in towards the pharynx. Sapid bodies have no apparent action on the anterior part of the organ, but they have an evident effect upon its centre and root. In dogs and cats, the lower jaw drops, after the intersection of the fifth pair on both sides, which greatly impedes deglutition, and sometimes renders it impossible. They have the same gait as rabbits, but instead of resting on the chin, they often press against the tongue which gets underneath, in consequence of the dropping of the jaw, and is rubbed against the ground during progression.

G. When only one nerve is cut, changes take place in the nostrils, mouth, and surface, of the larynx of the same side: half of the tongue becomes white, the epidermis becomes thickened, the gums separate from the teeth, and food lodges in the intervals thus formed; probably the animals being no longer arrested by feeling these substances, push them between the teeth and gums without being sensible of it.

H. Observation has led me to believe, *that the division of the fifth pair likewise entails the loss of hearing:* this would be less extraordinary, as in many animals the nerve of hearing is evidently only a branch of the trifacial.

*If this last result be correct, all the senses would thus be under the influence of the fifth pair, and the general theory of sensations would require to be reformed."*

---

*What are these experiments of M. Magendie's intended to prove?*

Their object, or rather the general inference from them, is simply this —that the olfactory nerve does not communicate the sense of smell, nor the optic nerve of sight, nor the auditory of hearing: but that the fifth pair communicates the senses of smell, and vision, and hearing, as well as of common feeling, or touch.

The proofs of this position are as follows:—With regard to smell, we find (section 1st,) that the olfactory nerves were laid bare, and a strong odoriferous caustic substance was applied to them without the animal manifesting any sensation: by which, as it appears to us, the simple fact of their insensibility to pain (a property common to all the anterior part of the brain) was proved, and nothing more.

We are next informed (section 2d,) that the olfactory nerve was entirely destroyed, the animal still retaining the sensibility to powerful odours, and having the same sensibility to the introduction of foreign bodies into the nostrils, as before the operation. This latter fact, having nothing whatever to do with the point in question, we shall not at present remark upon it. Let us observe the proofs that the animal retained the sense of smell. When liquid ammonia is held to the noses of rabbits, guinea pigs, or the other animals generally operated upon in these experiments, they turn away from it, and sometimes, particularly if any of it has touched the nose, rub their faces with the paws. They do so in those which have the olfactory nerve destroyed, as well as in those which have not. But does this prove that the irritation which they thus strive to remove is produced by the *smell* of the substances employed? To us there is no proof whatever that it does not proceed from the irritating nature of these bodies,— viz. liquid ammonia, strong acetic acid, and volatile oils, acting upon that kind of sensibility which the nostrils possess in common with other parts of the air-passages. If the same substances be held before the eye, they give rise to similar irritation; so likewise when inhaled into the larynx (section 4). Yet it is absurd to suppose that the *odour* of a body can affect either of these parts, although they are irritated by the causticity of these volatile substances.

The only fair experiment would be, to take bodies which have strong smell without being volatile or caustic; and such we may suppose their food to be to different animals. Accordingly, this was done by M. Magendie (section 6). Pieces of cheese and meat were rolled up in paper, and placed beside a dog, who tore off the paper, and devoured the contents. But observe, it is expressly stated that, when these were placed beside him in such a situation that he could not see them, they remained undiscovered. Nothing can more clearly show that he detected them by the sense of sight, and not by smell. It is very remarkable that these objections should be stated by M. Magendie himself (sections 5 and 6), apparently without his seeing their force.

The next proof offered is the converse of the preceding. The fifth pair was cut (section 4th); "and every trace of the action of powerful odours disappeared." That is to say, animals which before had turned away from ammonia, &c. did so no longer. If our reasoning on the preceding facts be correct, it will, *mutatis mutandis*, equally apply to these. The common sensibility being destroyed by the operation, the volatile particles of the ammonia, &c. coming in contact with the pituitary membrane, no longer excited any painful sensation; but we know of no means by which it could possibly be ascertained that the animals did not smell the substances, seeing that the sense of smell is not accompanied by any external manifestations. The eye, as well as the nose, was thus rendered insensible; but it is to be remarked, that the larynx remained sound, and was affected as before (section 4th). Is not this a strong proof that the mucous membrane of all these parts is equally irritated by the contact of acrid bodies, through the medium of their common sensibility?

The circumstances mentioned at section 7th, on the authority of M. Ramon, is altogether so loose and unsatisfactory, that we are surprised to find M. Magendie attaching any importance to them. We beg to state the following case, which is directly opposed to the observations of M. Ramon.—A patient applied some years ago at the Westminster General Dispensary, for complaints unconnected with the subject of this paper; but the circumstance in point is, that he bore the marks of a wound received by a musket-bullet, which had entered the os frontis on the median line, a little anterior to the coronal suture, and came out through the back part of the roof of the mouth.* The ball must necessarily have destroyed the first pair of nerves, in passing through the cribriform plate of the ethmoid bone; and, although there was no sinking of the nose, nor any visible destruction of that organ, so that abundant branches of the nasal division of the fifth pair must have remained, yet the sense of smell was entirely gone.—Here, then, was M. Magendie's experiment of destroying the olfactory nerves, performed on an animal having this superiority over those operated upon by him, that, being gifted with intellect, he was capable of giving the requisite information regarding a sense, viz. that of smell, which is not evinced by

* On writing to ask Mr. Hutchison if he remembered this patient, he returned the following answer:

"*Spring-Gardens; June 25.*

"Dear Sir,—I have received your note respecting the case of the man who was a patient of mine, at the Westminster General Dispensary, about six years ago, who had been wounded in the head by a musket-ball. I perfectly recollect the case, and have notes of it somewhere. But it may possibly serve your purpose for the present, if I state that the ball entered the cranium about the middle of the os frontis, appeared to me to have passed through the cribriform plate of the ethmoid bone, and escaped, or rather was cut out, from the posterior part of the palate. I perfectly recollect, also, that the man was subject to epileptic fits; *had lost the sense of smell*; that the wound was received at the Cape of Good Hope, near ten years previously to his consulting me; and, so struck was I with the case altogether, from its singularity, that I exhibited the man to the members of the Medical and Chirurgical Society, at one of their meetings.

Yours, truly,

*To Dr. Macleod.* A. C. HUTCHISON."

external manifestations; and this appears to us, under these circumstances, to be the only satisfactory means of judging.

Analogous to this case is the common observation, that caries affecting the upper part of the ethmoid bone destroys the sense of smell, though the patient continues to sneeze, and even to enjoy a pinch of snuff. Indeed, we have remarked, throughout this paper, that a due distinction is not made between the act or power of sneezing, and the sense of smelling. Sneezing is nothing more than a natural effort to throw off some irritating cause from the delicate membrane of the nose, just as coughing is to clear the throat. Now, if we destroy this sensibility by cutting the fifth pair, then we cannot by any means excite sneezing; a fact, of which none of our readers can be ignorant, who have perused the papers of Mr. BELL and Mr. SHAW. So entirely dependent is this act of sneezing upon the irritation of the fifth nerve, and so independent of the olfactory, that it is readily excited by tickling the nostrils with a feather, which is destitute of smell; while, on the other hand, the most powerful odours, whether pleasant or unpleasant, have no tendency to produce sneezing. Another fact tending to show that sneezing is independent of the olfactory nerve, is that it is sometimes excited by stimuli which do not act upon the nose at all. Thus, the writer of these remarks, by looking at the sun when it shines brightly, is made to sneeze as surely and as briskly as by taking snuff. And lastly, in common catarrh, the internal membrane of the nostril is frequently in such a high degree of excitement, as to be irritated by stimuli to which it is, under other circumstances, insensible. Thus, the mere exposure to the air will cause sneezing, while at the same time the sense of smell is almost lost. The only additional observation we have to make on this subject is, that we have numerous well-authenticated cases in which ossification of the falx, so as to press upon the olfactory nerves, has given rise to the most distressing sensations of smell.

Our remarks on the second paper will be extremely short, because our reasoning on the preceding, if good for any thing, in a great measure applies to both. The same operation on the fifth pair which renders the nostrils insensible to stimuli, likewise destroys the sense of touch of the other external parts, and, among the rest, of the eye; so that, when the cornea is rubbed, or even pricked, the animal manifests no symptom of pain. That this, however, has nothing to do with vision is proved, as well by the observations of Messrs. Bell and Shaw, as by the case related by Mr. CRAMPTON, of Dublin.* It is that of a young woman, who had so entirely lost the sense of touch all over the eyeball and neighbouring parts, as to suffer them to be handled without pain, or indeed any sensation; yet she saw distinctly with this eye.

The most important, and indeed the only, circumstance, in this paper, which renders the opinion that vision depends on the fifth pair plausible, is the contraction of the iris and its remaining insensible to light. When the experiment succeeds, this is always the case; and the pupil, indeed, is diminished almost to a point.

In addition to the circumstances mentioned by M. Magendie in his

---

* *Philosophical Transactions.* 1823.

paper, the following experiment was tried, at the suggestion of Dr. WOLLASTON :—A lighted candle was held before the eye, on the side where the nerve had been divided, with a view of ascertaining whether it would sympathetically produce any effect upon the sound eye. None resulted; and this was held to be an additional proof that vision was destroyed. The experiment, however, appears to us unsatisfactory, because, as we have already mentioned, the iris was so much contracted as to form almost a complete curtain between the retina and the light; and further, because, on applying the candle to the sound eye with the pupil of the natural size, some gentlemen thought the iris did contract, and some that it did not. We ourselves tried this experiment along with Dr. ELLIOTSON, and neither of us could perceive any contraction. In short, it was a matter of question whether, on applying a light before the eye of the sound side, the pupil contracted or not; which shows, at all events, that the degree of contraction, if any, was extremely small. Now, if the rays of light, having thus a free entrance through a natural and large pupil, produced so slight an influence upon the iris, what inference can we draw from the fact, that, when held before the other eye, in which the passage of the rays of light was almost entirely interrupted by the contracted iris, no sympathetic effect was produced in the other eye? Surely none at all decisive. This part of the argument proceeds upon the assumption that the iris and retina are mutually dependent upon each other, and that the movements of the former are an exact index of vision; but this is by no means universally the case. Some individuals are able to increase and diminish the pupil by a voluntary effort;* while, in some amorautic eyes, the iris contracts on the application of light, although the blindness be complete.

That vision is much impaired by this operation, we by no means deny: the appearance of the parts, as well as the gait of the animal (described in section E,) sufficiently show that it is so. But at the same time we would remark, that the eye is a very complicated piece of apparatus, which requires it to be in a perfect state in order to exercise its function; and a very curious fact, ascertained, among others, by M. Magendie himself, and mentioned in a preceding part of this Number, is that when, by any accident (as rendering the cornea opaque), this organ is brought into disuse, and its function no longer exercised, the optic nerve wastes. How can he account for this fact, if the optic be not the nerve of vision? Again, we protest against the accuracy of the experiment itself. That the fifth pair may be divided, is very possible; but that no other injury is done, seems not so easily proved. A sharp cutting instrument is thrust into the cranium, through the middle lobe of the brain, almost to the very centre of the base of the skull. The fifth pair lies so close by the cavernous sinus, that even Magendie, whose dexterity is unquestionable, cut it in the greater number of instances, so that the animals speedily died from effusion of blood. But besides this, the third, and more especially the fourth nerve, are here closely involved with the fifth; and, even if they be not actually divided, it is almost impossible but that they must be more or less injured

* This phenomenon occurs in the person of a distinguished member of the Medico-Chirurgical Society, and is particularly mentioned by Mr. SHAW in one of his papers.

and subjected to pressure, from the effusion of a portion of the blood, which must take place under the most favourable circumstances. Who, considering this, and having examined the anatomy of the parts in the rabbit and guinea-pig, will venture to argue from these experiments, as if it were certain that the fifth pair alone were merely and simply divided? "Il ne soit pas inutile," says M. Magendie, "de doubter quelque peu de choses les plus certaines."*

The circumstances with regard to the inflammation, and ultimate destruction, of the eye, are curious, and occurred exactly as described by M. Magendie, in the only rabbit which survived the operation: it lived a week. A phenomenon in strict analogy is presented by the hoof of the horse, which frequently becomes similarly affected from the division of the nerve.

The readers of this Journal must be aware that certain experiments have been performed on the nerves, and certain doctrines advanced concerning the nervous system in general, by Mr. CHARLES BELL and by Mr. SHAW. Now, as there are some points of resemblance, and some of difference, between their doctrines and those of M. MAGENDIE, it may not be irrelevant to point them out. Messrs. Bell and Shaw have cut the branches of the fifth pair after their exit from the cranium, and assert, as the result of numerous experiments, and lay it down as the basis of their doctrine, that this nerve communicates common sensibility, or the sense of touch, to all the parts whereon its branches are distributed. M. Magendie (who really writes and speaks as if he had not read the papers containing these researches,) cuts the fifth pair within the skull, and, like the gentlemen above mentioned, finds the parts supplied by its ramifications deprived of sensibility. So far he agrees with Mr. Bell and Mr. Shaw; but, while they rest at this conclusion, he goes further, and argues that smell and sight, and hearing likewise, depend upon the same nerve. With respect to hearing, we have no argument nor experiment brought forward in support of this opinion; and, therefore, we know not on what grounds it may be founded. With respect to the two former, we have endeavoured to show, that, however satisfactory his experiments may be, so far as they go, yet they are not such as to warrant the conclusions he has drawn from them.

---

* We understand, from Dr. J. SOMERVILLE, that several of the rabbits were afterwards examined by Mr. H. MAYO, who did not find any other nerve divided besides the fifth. Notwithstanding this, however, we submit, that the objections urged above are valid.

Besides the experiments above mentioned, M. MAGENDIE divided the fifth nerve as it passed along the fourth ventricle, in order to show its connexion with the medulla oblongata. The results were similar, but not so well marked, as when the division was performed higher up. Wherever the nerve was cut, its division was attended with very acute suffering, as evinced by the expressive cries of the animal. The uses of the posterior and anterior roots of the spinal nerves were very satisfactorily demonstrated on a young dog,—the division of the one set depriving the limb of motion, and that of the other of sensation. M. Magendie likewise performed the experiments, alluded to in the Historical Retrospect, of removing different portions of the brain; and with the effects there mentioned.—In case any of our readers should think of repeating these experiments, we have to warn them that their performance is attended with considerable difficulty; particularly the division of the fifth pair within the head, which frequently failed, even in Magendie's hands, from hemorrhage.

